# The Health of the Army

**Published:** 1908-08-29

**Authors:** 


### The Hospital

A Journal of

The Medical Sciences and Hospital Administration.

New Series. No. 77, Vol. III. [No. 1149, Vol. XLIV.] SATURDAY, AUGUST 29, 1908.

### The Health of the Army.

A distinguished member of the House of Cecil once wrote that "soldiers in peace are like chimneys in summer," and his words still reflect with tolerable accuracy the point of view of many civilians towards those whose business it is to fight for their country when occasion demands. But there are signs that the frame of mind, which draws a sharp distinction between the soldier in peace and the soldier in war, to the detriment of the former, is gradually becoming less common. Public opinion seems to be coming round to a saner and a more generous attitude towards the professional soldier and the profession of arms. This is shown in part by the closer attention which is now being paid to the problems of military organisation, and in part by the increasing interest which is being taken in the health and social welfare of the individual soldier. It is particularly satisfactory to note the gradual development which has taken place, and is still in progress, in the official recognition of the importance of the medical department of the Army. In former days the greatest and most successful generals were those who paid, so far as circumstances and their knowledge would allow, the greatest attention to the physical needs of their soldiers; but there is little indication in the annals of military history of a corresponding attitude at headquarters. Nowadays the importance of a healthy army is realised by those in authority, and in the military councils of every great Power there are signs of active systematic efforts towards the physical efficiency of troops in peace and in war.

In the Report of the Army Medical Department for 1907, Sir Alfred Keogh, the Director-General, submits to the Secretary of State for War an account of his stewardship during the past year. This official report on the health and sanitary conditions of the Army is distinctly encouraging, and the state of things which it represents is highly creditable to Sir Alfred Keogh and to the department which he controls with such zeal and foresight. The report says that, speaking generally, the health of the troops, both at home and abroad, was exceptionally good during last year. The hospital admissions, the mortality, and the invaliding and "constantly sick" rates were lower than in the preceding year, and showed a still further diminution when compared with the average for the decennial period 1897 to 1906. This decline in sickness has been general, and is not confined to any particular locality; but it has been most marked in India and in Egypt. The report draws especial attention to the remarkable improvement which has taken place in the health and morals of our troops in India during the period under review: the admission rate and mortality were both the lowest on record. A new departure was instituted last year in the method of invaliding soldiers stationed in India, and this piece of enterprise was attended by the happiest results. Instead of sending weakly men home to England, as had hitherto been done, the experiment was tried of despatching them during the cold season to selected hill stations, in whose magnificent climate they benefited to an astonishing degree. The result was a decrease of over a thousand invalids, as compared with the previous Indian trooping season. The report attributes much of the general improvement which has taken place in the health of the forces to the increased attention now paid to sanitation, not only by the medical authorities, but also by the soldiers themselves. This dawning appreciation, throughout all ranks of the Army, of the value of hygienic practices and of the risks which attend the neglect of sanitary precautions, inspires us with hope for an even more striking improvement in the future.

Since the stamping out of Mediterranean fever, by prophylactic measures based upon scientific research and reasoning, the health of our troops in Malta has undergone a wonderful change. In 1905 there were 643 admissions into hospital for this disease; last year there were only eleven. Encouraging results have also attended the widespread use throughout the Army of prophylactic injections of anti-typhoid vaccine. A subject which the report has every reason to draw attention to is the increasing prosecution of scientific research by officers of the Royal Army Medical Corps. The encouragement given by the authorities to this aspect of military medicine is a clear indication of the progressive spirit which now actuates the medical organisation of the Army. The victory over Malta fever, whose economic value to the Empire is now widely recognised, would alone justify the authorities in continuing to encourage laboratory investigations by every means in their power. So long as the Army medical department remains alert and progressive, we may feel confident that the debt which preventive medicine owes to the Royal Army Medical Corps will increase with each year that passes. The recent formation of a part-civilian part-military sub-committee of scientific experts, for the purpose of co-ordinating and directing the efforts of isolated workers in the field of medical research, has strengthened the Army Medical Advisory Board, and should be productive of valuable results. Nevertheless, although the scientific possibilities of military medicine are almost unbounded, we fear that, as long as our recruiting system continues to supply the Army with youths of poor constitution and degenerate parentage, so long will there be a limit to the endeavours of the Army Medical Service towards the improvement of the general physique of the British Army. The Director-General's report dwells with regret upon the poor physical condition of the average recruit, whom it takes two years to develop into the likeness of an able-bodied man. The physical test struggles hard to weed out the worst specimens, and a soldier's life is well suited to harden and develop the bodies and characters of those who are passed as fit; but even so, the situation is anything but satisfactory. The report, it is true, shows that in the proportion of admissions into hospital our Army compares favourably with those of the other great Powers, while in the matter of death-rate from all diseases our Army comes third of the six armies reported upon. Therefore when we read that "Average British recruits are not only the youngest, but in the poorest physical condition of those in any civilised army," we feel that, with such unpromising material to work upon, the administrative and medical authorities of our Army have done well to produce and maintain as good a general state of health as the figures indicate.

